# Effects of gum Arabic ingestion on body mass index and body fat percentage in healthy adult females: two-arm randomized, placebo controlled, double-blind trial

**DOI:** 10.1186/1475-2891-11-111

**Published:** 2012-12-15

**Authors:** Rasha Babiker, Tarig H Merghani, Khalifa Elmusharaf, Rehab M Badi, Florian Lang, Amal M Saeed

**Affiliations:** 1 Department of Physiology, Faculty of Medicine, University of Medical Sciences & Technology, P.O Box. 12810, Khartoum, Sudan; 2 Department of physiology, Faculty of Medicine, University of Tabouk, Tabouk, Saudi Arabia; 3 Department of Epidemiology and Public Health Medicine, Royal College of Surgeons in Ireland, P.O Box 15503, Adliya, Manama, Bahrain; 4 Department of Physiology, Faculty of Medicine, University of Khartoum, P.O Box 102, Khartoum, Sudan; 5 Department of Physiology, Eberhard-Karls, University of Tuebingen, Tuebingen, Germany

**Keywords:** Gum Arabic, Obesity, BMI, Body fat percentage

## Abstract

**Background:**

Gum Arabic (acacia Senegal) is a complex polysaccharide indigestible to both humans and animals. It has been considered as a safe dietary fiber by the United States, Food and Drug Administration (FDA) since the 1970s. Although its effects were extensively studied in animals, there is paucity of data regarding its quantified use in humans. This study was conducted to determine effects of regular Gum Arabic (GA) ingestion on body mass index and body fat percentage among healthy adult females.

**Methods:**

A two-arm randomized, placebo controlled, double-blind trial was conducted in the Department of Physiology at the Khartoum University. A total of 120 healthy females completed the study. They were divided to two groups: A test group of 60 volunteers receiving GA (30 gm /day) for 6 weeks and a placebo group of 60 volunteers receiving pectin (1 gm/day) for the same period of time. Weight and height were measured before and after intervention using standardized height and weight scales. Skin fold thickness was measured using Harpenden Skin fold caliper. Fat percentage was calculated using Jackson and Pollock 7 caliper method and Siri equation.

**Results:**

Pre and post analysis among the study group showed significant reduction in BMI by 0.32 (95% CI: 0.17 to 0.47; P<0.0001) and body fat percentage by 2.18% (95% CI: 1.54 to 2.83; P<0.0001) following regular intake of 30 gm /day Gum Arabic for six weeks. Side effects caused by GA ingestion were experienced only in the first week. They included unfavorable viscous sensation in the mouth, early morning nausea, mild diarrhea and bloating abdomen.

**Conclusions:**

GA ingestion causes significant reduction in BMI and body fat percentage among healthy adult females. The effect could be exploited in the treatment of obesity.

## Introduction

Gum Arabic (GA) is derived from exudates of Acacia senegal or Acacia seyal trees. It consists of a mixture of polysaccharides (major component) plus oligosaccharides and glycoproteins [1,2]; however, its composition can vary with its source, climate and soil. Sudan is the world′s largest producer, followed by many other African countries. It readily dissolves in water to form solutions characterized by low viscosity. This allows its use in various applications [3]. It is used as an emulsifier, thickening agent and flavor stabilizer in both the pharmaceutical and food industries. It is also used in textile, pottery and cosmetics industries. The FAO/WHO Joint Expert Committee for Food Additives defined it as a dried exudation obtained from the stems of A. Senegal or closely related species of Acacia [4].

Gum Arabic was evaluated for acceptable daily intake for man by the Joint FAO/WHO Expert Committee on Food Additives since 1969 [5]; however, Sudanese people in Western Sudan had been using it for long time without limitations. It is indigestible to both humans and animals, not degraded in the intestine, but fermented in the colon to give short-chain fatty acids, leading to a large range of possible health benefits [6]. One of these benefits is its prebiotic effect [7,8]. It has been claimed that four week supplementation with Gum Arabic (10 g/day) led to significant increases in Bifidobacteria, Lactobacteria, and Bacteriodes indicating a prebiotic effect [8]. Other effects include reduction in plasma cholesterol level in animals and humans [9], anticarcinogenic effect [10] and anti-oxidant effect [11,12] with a protective role against hepatic and cardiac toxicities. In addition to that, it has been claimed that Gum Arabic alleviates effects of chronic renal failure in humans; however, further studies are needed for confirmation [13-15].

Several epidemiological studies suggest that a high intake of dietary fiber, including GA, is associated with beneficial effects on fat metabolism [14,16]. Dietary fiber promotes satiation and satiety, alter glycaemic index, affects gastric emptying, gut hormone secretion and thus helps to manage weight [17]. Leptin promotes weight loss by two different mechanisms. It reduces appetite, and thus food intake, and at the same time increases energy expenditure also dietary fiber was inversely associated with leptin level in young Japanese adults [18,19]. In addition to that, a study has shown that GA inhibits intestinal glucose absorption via interaction with membrane abundance of SGLT1 in mice [20].GA significantly blunted the increase in body weight, fasting plasma glucose and fasting insulin concentrations during high fat diet.

Obesity is a well known risk factor for coronary heart disease, stroke, diabetes and many other abnormalities, including cancer [21,22]. These complications depend not only on absolute amount of fat but also on its distribution. Absolute total body fat and adipose tissue distribution are known to be associated with cardiometabolic risk in adult females [23]. At least in theory, Gum Arabic can serve to reduce obesity and therefore prevent associated complications in humans. The aim of this study is to determine the effects of Gum Arabic ingestion on weight, body mass index and body fat percentage among healthy adult females in randomized, placebo controlled and double-blind study.

## Methods

This is a two-arm randomized, placebo controlled, double-blind study comparing an intervention group receiving 30 gm of GA daily for 6 weeks with a control group receiving a placebo for the same period of time. The study was conducted at the Department of Physiology, Faculty of Medicine at the University of Khartoum during the period from April to July 2011. All participants were female students from the University. Inclusion criteria were age 17 years or above and healthy with no symptoms or signs of acute or chronic medical illness. Exclusion criteria were age less than 17 years, past or present history of metabolic, gastrointestinal, degenerative and/or inflammatory diseases, smoking, drug abuse or alcohol consumption, use of corticosteroids or any other drug that affects body weight, and history of Gum Arabic (GA) allergy. Participants were asked to take habitually daily diet and to avoid exercise during the period of the study.

To detect a reduction in body weight of 2 kilograms (SD 4 kilograms), with a two-sided 5% significance level and a power of 80%, a sample size of 60 subjects per group was calculated based on normogram for comparison of means in two equal sized groups [24]. Random allocation was achieved by generating series of numbers by independent third-party not associated with the study. Sealed boxes were prepared containing supplements package of either intervention (Gum Arabic) or placebo (pectin). After the randomization sequence generated the boxes containing the supplements was given to the blinded investigator responsible for enrollment. Follow up assessments were undertaken by the chief investigator who was also blinded to the randomization.

**Figure 1:**
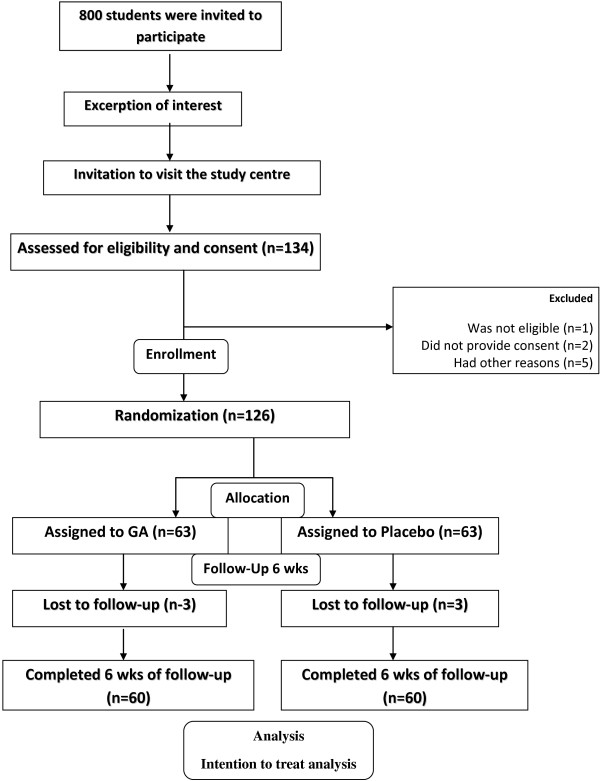
Follow of participants throughout the intervention.

Eligible participants were 120 students. They were all enrolled and randomly allocated into either intervention (n= 60) or placebo (n= 60) group by the blinded investigator (Figure 1). Each participant was supplemented with a daily dose of either Gum Arabic or a placebo. The dose of Gum Arabic was 30 g of 100% natural gum provided in a powder form by ″Dar Savanna Ltd. Khartoum, Sudan″. Its quality was consistent to the requirements of Food and Agriculture Organization of the United Nations (FAO) and British pharmacopoeia (BP). The dose was divided in 5 sachets each containing 6 grams, consumed in two divided doses; early morning dose of 3 sachets (18 gram) and evening dose of 2 sachets (12 gram) four hours after meal. The dose of placebo was 1 g of pectin given in two divided doses in the same way. Each dose was reconstituted in 250 ml of water and shaken well to ensure adequate mixing before intake.

Weight was measured by using digital physician′s scale to the nearest 0.1 kg. Height was measured by using calibrated physician′s scale to the nearest 1 cm. BMI was calculated by the standard formula: weight (kg) / height (m) ^2^.

Skin-fold thickness was measured using a harpenden Skin fold caliper. It was measured at 7 different anatomical sites (chest, axilla, triceps, subscapular, abdomen, suprailium, and thigh skin folds (mm). Three sets of measurements were averaged for each site. The following Jackson- Pollock formula was used to calculate body density (BD) [25]. BD = 1.11200000 − 0.00043499(X) + 0.00000055(X) (X) − 0.00028826(A) (where X = Sum of chest, axilla, triceps, subscapular, abdomen, suprailium, and thigh skin folds in millimeters and A = age in years). Then the Siri equation was used for calculation of body fat percentage from body density (*%* Fat = [(4.95/BD) − 4.5]100 [26].

Institutional review board approved this study. Appropriate written consents were obtained from each student before enrollment in the study. All data were collected prospectively by the researchers at the department. Double data entry and cross validation were employed to ensure validity and quality of data. Data were analysed using STATA-10 program. The paired t test was used for analysis of pre and post-intervention data. The independent sample t test was used for comparison between the intervention and control groups. A p-value of less than 0.05 was considered statistically significant.

## Results

**Figure 2:**
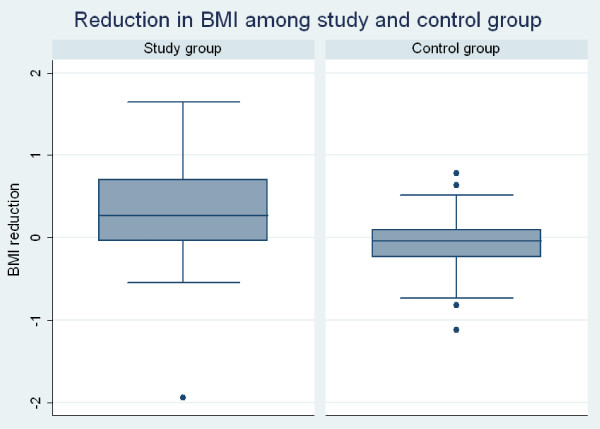
Reduction in BMI among study and control group.

**Figure 3:**
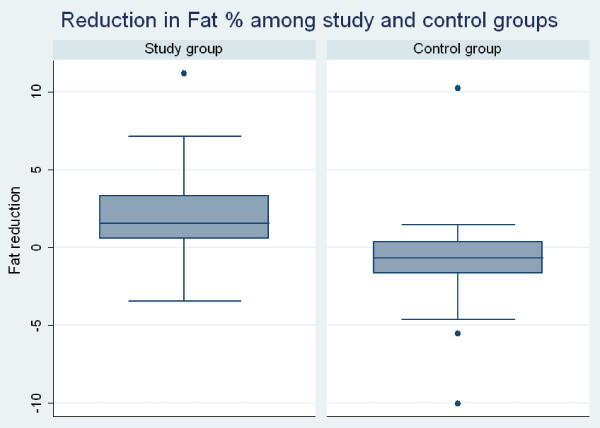
Reduction in fat % among study and control groups.

Characteristics of the participants are shown in (Table 1). Pre and post analysis among the study group showed weight difference % of 1.24 from 72.25 kg to 71.43 kg ± 1.94 (mean ± SD) within the study group and minor increase of weight from 68 kg to 68.19 kg ± 1.35 (mean ± SD) (Table 2). A significant reduction in BMI (Figure 2) by 0.32 from (mean ± SD) 27.31 ± 5.4 to 26.99 ± 5.54 (95% CI: 0.17 to 0.47; P<0.0001) and body fat percentage (Figure 3) by 2.18% (95% CI: 1.54 to 283; P<0.0001) following regular intake of 30 gm /day Gum Arabic for six weeks (Table 3). Ingestion of the placebo caused significant increase in the percentage of body fat from (mean ± SD) 18.31 ± 4.14 to 19.13 ± 4.06 (95% CI: -1.44 to −0.20; p = 0.010) and tended to increase BMI from (mean ± SD) 25.78 ± 3.85 to 25.85 ± 3.80 (95% CI: -0.16 to 0.02; p = 0.132), an effect, however, not reaching statistical significance (Table 3). Side effects caused by GA ingestion were experienced only in the first week. They included unfavorable viscous sensation in the mouth, early morning nausea, mild diarrhea and bloating abdomen (Table 4).

**Table 1:** Characteristics of cases in the study and control groups

**Parameter**		**Minimum**	**Maximum**	**Mean**	**SD**	**P value**
Age (years)	Study group	17	31	19.37	1.97	0.670
	Control group	18	35	19.53	2.25	
Height (m)	Study group	1.50	1.85	1.63	0.064	0.620
	Control group	1.53	1.73	1.62	0.057	
Weight (kg)	Study group	54.30	121.20	72.30	13.26	0.054
	Control group	46.30	95.50	68.01	10.78	
BMI (kg/ m^2^)	Study group	17.53	51.10	27.31	5.42	0.076
	Control group	17.22	34.66	25.78	3.85	
Body fat %	Study group	11.70	32.00	19.45	4.24	0.14
	Control group	9.50	26.90	18.31	4.14	

**Table 2:** The Percent changes on Body Weight among Study and Control groups

**Weight (kg)**		**Mean**	**Mean difference%**	**95% Cl (LL,UL)**	**SD**
(study group)	Before	72.25	1.24	- 0.70, 3.18	1.94
	After	71.43			
(control group)	Before	68.00	**-** 0.31	**-** 1.66, 1.04	1.35
	After	68.19			

**Table 3:** Body mass index and body fat percent changes among study and controls

**Parameter**		**Min**	**Max**	**Mean**	**SD**	**Mean Difference**	**P value**
BMI (study group)	Before	17.53	51.10	27.31	5.42	−0.32	0.0001
	After	17.36	53.04	26.99	5.54		
BMI (control group)	Before	17.22	34.66	25.78	3.85	0.07	0.132
	After	17.40	34.62	25.85	3.80		
Fat % (study group)	Before	11.70	32.00	19.45	4.24	−2.18	0.000
	After	9.49	30.50	17.26	4.28		
Fat % (control group)	Before	9.50	26.90	18.31	4.14	0.82	0.010
	After	10.53	25.90	19.13	4.06		

**Table 4:** Side effects of intervention among study and controls

		**Study group (n=60)**		**Control group (n=60)**		**P value**
		**n**	**%**	**n**	**%**	
Nausea	Yes	49	81.7%	0	0.0%	0.000
	No	11	18.3%	60	100.0%	
Diarrhea	Yes	54	90.0%	11	18.3%	0.000
	No	6	10.0%	49	81.7%	
Unfavourable oral viscous sensation	Yes	60	100.0%	0	0.0%	0.000
	No	0	0.0%	60	100.0%	
Bloating abdomen	Yes	9	15.0%	0	0.0%	0.002
	No	51	85.0%	60	100.0%	

## Discussion

The results showed that regular intake of 30 gm /day GA for six weeks resulted in significant reduction in BMI and body fat percentage (P<0.0001) ,Changes in body weight were reported to occur with many other fibers intake whether the fiber is obtained from naturally high-fiber diet or when it is ingested in a form of a supplement [27].

The US Food and Drug Administration consider Gum Arabic (GA) as one of the safest dietary fibres [28]. In this study 60 healthy female volunteers consumed GA without doubt as many Sudanese used to ingest GA for both health benefits and nutritional purposes. Females were selected as they are more concerned with aspects of their apperarance, particularly weight [29]. The effects of GA on BMI and fat percentage were studied among these females.

Gum Arabic consumption seems to be an effective dietary strategy to prevent or treat overweight with its several biological mechanisms [17], Obesity is a worldwide problem that is associated with many complications. Even though regular exercise and dieting are effective and non-invasive measures used for its treatment, compliance to these measures is limited [30]. The role of dietary fibers in prevention and treatment of obesity has been studied in both humans and animals [27,31]. Although Gum Arabic influence on energy intake and body weight regulation remains controversial. A growing body of scientific evidence indicates that GA ingestion causes significant reduction in caloric intake with an increased subjective feeling of satiety [32].

Many studies suggested a strong positive correlation between blood leptin concentration, BMI and intake of dietary fiber, On the other hand, serum leptin concentrations were not related to dietary patterns in the US population [33] and no significant correlation was found between leptin and dietary fiber [34].

In addition to these effects, dietary fibers including GA bind bile acids and diminish their absorption in the terminal ileum [35]. Then in the large intestine, degradation of GA releases the sequestered bile acids and the acidic pH generated during the fermentation process renders them insoluble and promotes their excretion in stool [35]. This reduces their pool in the body and causes decreased fat digestion and absorption. Similarly, the hepatic formation of new bile acids requires cholesterol. Thus, prolonged ingestion of Gum Arabic may cause weight loss and reduction in cholesterol level in plasma

In our study the effect reflected by a reduction in body weight by 1.24% from 72.25 to 71.43 ± 1.94 (mean ± SD) within the study group. A recent proposed mechanism by which viscous dietary fibers were found to preserve lean body mass and reduce adiposity is increased mitochondrial biogenesis and fatty acid oxidation by skeletal muscles [36]. Gum Arabic mechanism is not yet fully elucidated, because of a small number of conducted studies. This study highlights the effect of gum Arabic on BMI and fat %; it would be wise to conduct a long-term studies, evaluating complete range of parameters with different groups and doses to elucidate the mechanism of action of GA on reducing obesity and its prevention.

Previous studies have shown that a daily dose of 30 g of GA can be tolerated by most subjects and the main complaint was excessive flatulence [37]. However, this complaint was found to be mild, even at doses >50 g/day. In our study symptoms were only experienced in the first week of supplementation and disappeared later. Unfavorable viscous sensation in the mouth was the main complaint; however, addition of a flavor to GA solution, as practiced by many of the volunteers, was found to be useful. Diarrhea which was reported by 90% of cases could be the result of increased intestinal motility due to the increase in bulk of stool. It is worth noting that previous studies described GA as a treatment rather than a cause of diarrhea [38].

One of the limitations in our study is not measuring blood leptin concentration, due to resource limitation. Another major limitation in this study is the high dose of GA ingested daily by students in the group of cases compared with the low dose of the placebo taken by the controls. However, our results do confirm that regular ingestion of GA causes significant reduction of body mass index and body fat percentage among subjects. This effect can be considered for treatment or prevention of obesity.

## Conclusions

Gum Arabic ingestion causes significant reduction in BMI and body fat percentage among healthy adult females. The effect could be exploited in the treatment of obesity.

## Competing interests

The authors declare that they have no competing interests.

## Authors’ contributions

RB has made enrolment and random allocation of participant, acquisition of measurements and data, followed the study and drafted the manuscript. THM participated in the sequence alignment, coordination and helped to draft the manuscript. KE designed and revised the methodology, statically analyzed the data and revised the manuscript. RMB generated the Idea and participated in designing the protocol and follow up. FL has been involved in revising it critically for important intellectual content, drafted and revised the manuscript. AMS made contributions to conception and design, directed the study, drafted and revised the manuscript. All authors read and approved the final manuscript.
